# The Follow-Up of Eating Disorders from Adolescence to Early Adulthood: A Systematic Review

**DOI:** 10.3390/ijerph192316237

**Published:** 2022-12-04

**Authors:** Caterina Filipponi, Chiara Visentini, Tommaso Filippini, Anna Cutino, Paola Ferri, Sergio Rovesti, Emanuela Latella, Rosaria Di Lorenzo

**Affiliations:** 1 School of Nursing, Department of Biomedical, Metabolic and Neural Sciences, University of Modena and Reggio Emilia, 41125 Modena, Italy; 2 Service of Psychiatric Diagnosis and Care (SPDC), Department of Mental Health and Drug Abuse, AUSL, 41126 Modena, Italy; 3 Environmental, Genetic and Nutritional Epidemiology Research Center (CREAGEN), Section of Public Health, Department of Biomedical, Metabolic and Neural Sciences, University of Modena and Reggio Emilia, 41125 Modena, Italy; 4 School of Public Health, University of California Berkeley, Berkeley, CA 94704, USA; 5 School of Psychiatry, University of Modena and Reggio Emilia, 41125 Modena, Italy; 6 Department of Biomedical, Metabolic and Neural Sciences, University of Modena and Reggio Emilia, 41125 Modena, Italy

**Keywords:** anorexia nervosa, bulimia nervosa, adolescence, eating disorders, medical and psychiatric comorbidity, substance use, social–relational complications

## Abstract

Eating disorders (EDs) are common among children and adolescents and are characterized by excessive concerns for physical appearance, distorted body image, and fear of gaining weight. The purpose of this review is to evaluate the follow-up of EDs from adolescence to adulthood, analyzing persistence, relapses, and associated comorbidities. We searched scientific articles in PubMed, PsycInfo, Scopus, and Embase through two research strings, one for quantitative outcomes (recovery/persistence, relapse, and remission) and one for the other outcomes (psychiatric and medical comorbidities, substance use, and social–relational complications). From a total of 8043 retrieved articles, we selected 503 papers after exclusion of duplicates and title/abstract screening. After a full-text evaluation, we included 16 studies eligible for this review. We performed a meta-analysis describing the quantitative results, and we created a narrative synthesis for the qualitative outcomes. Results: Our results confirm that EDs can persist in early adulthood in 40.7% of cases with a relapse percentage of 24.5%. Individuals with an ED more frequently present with an empathy deficit and comorbid anxiety and depressive disorders. EDs are chronic and complex disorders, more frequent in females. In most cases, EDs reduce the autonomy of individuals who present many difficulties in affirming their independence from parental family.

## 1. Introduction

Anorexia nervosa (AN) and bulimia nervosa (BN) are eating disorders (EDs) characterized by severe distortion of the body image, excessive concern for physical appearance, and extreme fear of obesity or weight gain [1]. The first documented case of anorexia nervosa was reported in 1888 in *The Lancet*. In this article, the author describes the conditions of a fourteen-year-old girl, who was extremely underweight and visibly emaciated and refused to eat anything for no apparent reason [2]. Bulimia nervosa was identified between the 1930s and 1940s as a combination of “food reversals” and self-induced vomiting, linked to severe weight gain and a distorted body image [3].

According to the criteria of the ICD-10, the disorder of anorexia nervosa is characterized by a deliberate loss of weight, which is associated with a specific psychopathology, including the fear of gaining weight [4]. Bulimia nervosa is defined as a disorder characterized by repeated episodes of overeating and an obsession with body weight, which leads to the occurrence of excessive food ingestion followed by vomiting or use of laxatives [4].

Throughout the several versions of the *Diagnostic and Statistical Manual of Mental Disorders* (DSM) [5,6,7,8,9,10], different diagnosis criteria for anorexia nervosa have been adopted, in particular regarding the following symptoms: amenorrhea, the percentage of weight loss in a given time, or the patient’s awareness toward the disease.

We observed an evolution of the DSM diagnostic criteria, the latter of which allow us a more effective and inclusive diagnosis than the previous ones. Sunday’s study (2001) applied the DSM-III-R and DSM-IV criteria to the same patients and found that only six diagnoses could be made by applying the DSM-III-R criteria, while the number of diagnoses increased to 14 with the DSM-IV criteria [11].

Epidemiological research on eating disorders is quite complex due to the lack of uniformity of studies and the modification of diagnostic criteria over time. Moreover, epidemiological data change based on geographic location. In Latin America, the AN prevalence is 0.1%; the bulimia prevalence is 1.16%, and binge eating disorders are (BEDs) 3.53% [12]. In Europe, AN and BN present a similar prevalence, ranging from 1% to 4% for anorexia and 1% to 2% for bulimia nervosa [13]. In the United States, American Psychiatric Association (APA) indicates a prevalence of anorexia between 0.5 and 3.7 percent in the female population and between 1.1 and 4.2 percent for bulimia [14]. In Italy, the prevalence of people affected by anorexia nervosa ranges between 0.2% and 0.8%, whereas the prevalence of bulimia ranges between 1% and 5% [14]. In the UK, a recent study on people 8–17 years of age reported an ED incidence of 13.86 persons per 100,000 inhabitants [15].

Regarding the gender difference, a recent study highlights that the lifetime prevalence rates of anorexia nervosa might be up to 4% among females and 0.3% among males, whereas regarding bulimia nervosa, the rates are up to 3% in females and more than 1% in males [16].

ED causes are controversial because many genetic [17,18,19], biological [20,21], psychological, and social factors can contribute to the development of these diseases [22,23].

Regarding the psychological factors, the personality trait of alexithymia has been associated with the development of EDs. The alexithymic subject is not able to recognize nor describe his/her own feelings and has many difficulties in empathizing with the others, showing compensatory behavior, such as obsessive eating [22]. Other psychological traits, often observed in patients affected by EDs are poor coping strategies and low self-esteem, also associated with a very poor psychosocial outcome [23]. The family environment can greatly contribute to the development of EDs, especially during adolescence [23]. The presence of an obese family member with correlated health problems can induce the fear of becoming obese in adolescents, who, as a reaction, can implement strict weight loss diets to control body weight. This behavior can be read as an attempt to avoid self-identification as a family member [24]. Although dietary regime is rarely associated with EDs, it still represents a risk factor [25]. The intergenerational transmission of dietary patterns plays a role in EDs as suggested by some authors who analyzed the relationship between parental feeding practices and children’s eating problems [26]. A recent study has highlighted that direct parental feedback on child eating, weight, or shape, such as encouragement to diet and criticism of weight or shape, can induce potentially negative outcomes, including eating disorders [27]. In this regard, the American Academy of Pediatrics has recently released recommendations to prevent both eating disorders and obesity among children and adolescents [28].

From the historical observations of Minuchin et al. [29] and Selvini Palazzoli [30], family dynamics alone can foster the development and maintenance of EDs, in particular of AN, due to distorted, rigid, and confounding intrafamilial relationships. For many years now, family therapy according to the Maudsley method has been recommended for families with a member affected by EDs [31], and group educational interventions are indicated for parents distressed and burdened by symptoms and behaviors of EDs [32].

### 1.1. Clinical Course of Eating Disorders

The definitions of relapse, remission, and recovery are essential for a clinical investigation of an ED, which can become chronic over time and can be associated with psychiatric comorbidities and organic or social complications and cause death due to medical complications or suicide. Nevertheless, there are no specific criteria available for defining a patient with an ED as recovered or remitted, but there are multiple and differing criteria due to the difference in study design and the changes in diagnostic criteria over the years. In this perspective, Kahlsa and his collaborators performed a systematic review in 2017 subdividing the literature into three main categories according to the definitions of remission and recovery based solely on weight data, symptoms, or both weight and symptoms combined [33]. They reported that relapse rates ranged between 9 and 52%, which tended to increase with the increasing duration of follow-up and which was especially high within the first year following treatment in persons with AN [33].

For EDs, the historical classification of Morgan and Russell [34] is still valid and often cited in the literature and used in clinical practice, according to whose clinical evaluation the following specifications are defined: “Good” indicates the return to regular menstrual cycle and maintenance within 15% of the average body weight; “Intermediate” indicates the maintenance of 15% of the weight but without constancy over time and alteration of the menstrual cycle; “Poor” includes weight less than 15% of the average, a symptom that causes concern in the patient or those close to her, and absent or highly irregular menstrual cycle; “Died” due to fatal ED complications.

### 1.2. Remission and Relapse

To better explain the different interpretations given to remission and relapse in EDs, we can refer to the studies by Lowe et al. [35] and Kordy et al. [36], both including the concept of partial remission. Lowe et al. define as partially remitted the patient who obtains a score between 2 and 4 on the Psychiatric Status Rating (PSR) [37], a scale used to assess the degree of mental illness, within the above-mentioned criteria of Morgan and Russell to evaluate the outcome [34]. Kordy et al. [36] analyze the clinical course of ED according to the body mass index (BMI) modifications. The subject with restrictive anorexia nervosa is considered in partial remission when he/she reaches a BMI of 17.5; in the case of bulimia with compensatory behavior, abstention from both self-induced vomiting and laxative abuse and a maximum of one binge episode per week are required to define a partial remission.

For the definition of complete remission in EDs, some studies suggest precise indicators:

(1) BMI > 17.5, regular menstrual cycle, and no diagnosis of ED [37];

(2) BMI > 19, fear of weight gain should not be present, and compensatory symptoms, such as self-induced vomiting or laxative abuse, should not occur for at least 12 weeks [36];

(3) A total of 1 or 2 residual symptoms or a total absence of symptoms, with a PSR score less than or equal to 2 [38]. Regarding relapse, the studies by Fichter and Quadflieg [39] and Eisler et al. [40] identify relapse with the “Poor” outcome according to the scale of Morgan and Russell [34]. For other authors, Herzog et al. [41], Keel et al. [38], and Helverskov et al. [42], the relapse of an ED indicates the presence of a new clinical condition of “full symptoms” and/or a PSR scale score of between 5 and 6. According to Kordy et al. [36], relapse can be defined as a change from a state of partial or complete remission by the symptomatology described in the DSM-IV.

The study by Walsh et al. [43] presents the broadest criteria for the definition of relapse: a BMI less than 16.5 for at least two consecutive weeks correlated with medical complications, risk of suicide, or development of an additional psychiatric disorder, which requires treatment.

In the literature, recovery is very frequently attributable to the “good” outcome of the Morgan and Russell rating scale and to the disappearance of compensatory symptoms, more frequently typical of bulimia nervosa [34]. In the most recent studies, the criterion of amenorrhea is not widely considered because the association between body weight and menstrual cycle absence has not been scientifically proven.

### 1.3. Comorbidities and Complications

Psychiatric complications are usually associated with the acute phase of EDs [44]. According to a study conducted by Micali et al. [45] on a sample of adolescents with anorexia nervosa, the most frequent comorbid disorders in this age group are depression and anxiety disorders. Another study, carried out on a large Swedish adult sample, evaluated mental disorders in patients who had already been diagnosed with an ED, highlighting that the diagnoses with the highest number of cases were mood disorders and anxiety disorders. In particular, major depressive disorder affected the greatest number of individuals with EDs than all other psychiatric comorbidities. Among the anxiety disorders, the most reported was generalized anxiety, followed by social and specific phobia, obsessive compulsive disorder, post-traumatic stress syndrome, and episodes of panic attacks. In another study, alcohol and drug addictions have also been reported [46].

Other authors highlighted that borderline personality disorder can be associated with EDs, contributing to create the background that participates in shaping the ED evolution [47]. In particular, one study pointed out that eating disorder not otherwise specified can represent a separate cluster of eating disorders among borderline women, rather than a prodromal or residual form of anorexia or bulimia nervosa [48].

Medical complications are primarily the result of malnutrition and rapid weight loss or can be caused by compensatory behaviors. In accordance with a recent review, medical comorbidities complicate 42% of ED cases [44]. They cause deficits at different levels of systems: in the integumentary system, symptoms, such as dry skin, hair loss, nail fragility, formation of pressure ulcers in conjunction with the bone protuberances [49], or acrocyanosis, a bluish discoloration of the skin caused by hypothermia and impaired blood circulation [50]; at the gastrointestinal level, there is abdominal pain, nausea, constipation, and bloating as a result of slow intestinal motility [51]; at the muscle level, there is a loss of skeletal muscle tissue and reduced strength [52]; for the hepatic system, liver disease can manifest itself in rapid and massive weight loss [53]; in the cardiovascular system, there are cardiovascular complications due to a reduced perfusion, and, moreover, pericardial effusion can occur [54] as well as a prolapse of the mitral valve [55]. In eating disorders, the medical complications can be perforation of the stomach after acute dilatation, multiple suicide attempts, aspiration, injury or rupture of the esophagus, severe bleeding from the rectum causing anemia due to laxative abuse, hypokalemic nephropathy, depressive disorders due to starvation, and severe erosion of the enamel of the teeth resulting in extensive loss of teeth [56].

The studies that investigated social complications in EDs show that a common feature of patients suffering from eating disorders is the difficulty in creating social networks, due to a basic shyness especially typical of anorexia nervosa, and the inability to establish solid connections with one’s family or peers. In a qualitative study, adolescents with EDs reported persisting social difficulties in developing and maintaining social networks and interpersonal skills [57].These difficulties in establishing satisfactory interpersonal relationships lead the subjects affected by EDs to a reduced search for social stimuli and minimal sensation of pleasure in joining in social contexts [58]. Contemporary models of eating disorders have highlighted that both cognitive style and social emotional difficulties are involved in the maintenance of EDs [59]. Another study, focused on ED biopsychosocial outcomes, highlighted feelings of dissatisfaction and difficulties relating to the sexual sphere probably related to social phobia, low self-esteem, and uncertain definition of social identity [60].

### 1.4. Purpose

To better evaluate the maintenance of eating disorders in a crucial period of life, such as early adulthood, this systematic review is aimed at assessing the persistence, remission, and relapse of EDs and related comorbidities and/or social–relational complications in the transition period from adolescence to early adulthood.

## 2. Materials and Methods

We performed this systematic review through the following steps: formulation of the research questions, criteria for identification studies, literature search, qualitative analysis of studies collected, statistical analysis of quantitative data, and quality evaluation of the selected studies.

### 2.1. The Research Questions

The research questions were the following:

(1) What is the percentage of ED persistence from its onset to early adulthood considering remission, relapse, and death?
(2) What kinds of psychiatric, medical disorders, and/or substance use comorbidities are associated to EDs in early adulthood?
(3) What kinds of social–relational difficulties can complicate EDs in early adulthood?

### 2.2. Study Identification

**Figure 1:**
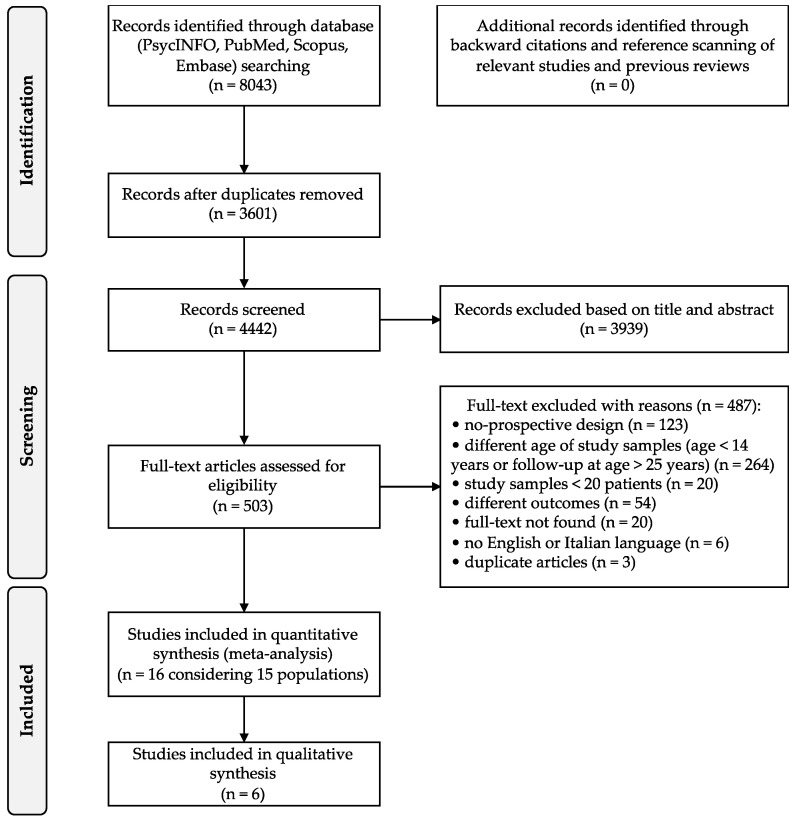
PRISMA flow diagram.

We adopted the PRISMA flow diagram [61,62] to describe the sequence of steps (identification, screening, eligibility, and inclusion) for the collection and identification of eligible studies, as shown in Figure 1.

Following the formulation of the research questions reported above, we selected the keywords, which were entered into the PsycInfo, PubMed, Scopus, and Embase electronic databases.

### 2.3. Inclusion Criteria

The criteria of inclusion and exclusion were defined in accordance with the research questions in order to limit and focus the research on the elements of interest. We took into account only articles written in English or Italian, studies with prospective design, and with samples of at least 20 patients aged between 14 and 25 years with ED occurrence before age 18. There were no limitations regarding the publishing year, countries, or environment setting. Other systematic reviews were used to research and conduct an in-depth analysis of the topic of this systematic review.

### 2.4. Literature Search

During the screening phase, we did not include limits about the studies’ countries and time of publishing; this permitted us to analyze a wide time window, from 1985 to 2021, in different geographical zones although only in Western countries.

We identified studies by applying the following search strings intertwined by the use of Boolean operators on four different databases that were PsycInfo, PubMed, Scopus, and Embase up to 30 September 2021:

− (Anorexia OR bulimia OR eating disorder) AND (psychiatric comorbidity) AND (substance use);
− (Anorexia OR bulimia) AND (outcome OR follow-up) AND (recovery OR relapse).

In the articles considered according to our inclusion criteria, we extrapolated the data of interest, such as information on the country and year of publication, study methodology, follow-up period, and sample data (number, female percentage, ED type, age at onset and at follow-up, medical and psychiatric comorbidities, substance use, social–relational complications, and pharmacological treatments). In the selected studies, we analyzed our outcomes according to the research questions, performing a quantitative analysis on ED persistence and relapses in early adulthood, and ED mean duration. We described and summarized in a narrative synthesis, performing a qualitative analysis, the other outcomes of psychiatric and medical comorbidities, substance use, and social–relational complications.

To avoid bias, a multiprofessional team cooperated in selecting articles and analyzed data independently, discussing with each other in case of discrepancies, in accordance with review authors’ indications [63]. After the selection phase, we analyzed data from the selected articles concerning the number of individuals in each sample, the drop-out rate, the female percentage, the mean age at baseline and follow-up, the specific eating disorder, and if the patient was in treatment (either pharmacological, psychotherapy, or multimodal) or not.

### 2.5. Statistical Analysis

The weighted mean (WM) was calculated with its corresponding confidence interval (CI) at 95% for the quantitative analysis: percentage of persistence, relapse, and ED length. The dimension of each study population was used as weight, either considering it in its entirety or on a subgroup using metaprop command of STATA software (v17, StataCorp., College Station, TX, USA, 2021).

### 2.6. Study Quality Assessment

We used the Newcastle–Ottawa Scale (NOS) to assess the quality and pertinence of the articles included in this Systematic Review [64]. Each study could reach a maximum of 9 points based on selection, comparability, and outcome for cohort studies and selection, comparability, and exposure for case–control studies.

## 3. Results

This section is divided by subheadings and provides a concise and precise description of the experimental results, their interpretation, and the experimental conclusions that can be drawn.

### 3.1. Study Selection

Initially, we found 8043 articles, which were downloaded in their entirety and imported on Mendeley, which allowed us to facilitate the study, handling, and further screening of the articles. The process is illustrated in a PRISMA flow chart shown in Figure 1. In accordance with our inclusion and exclusion criteria, we removed 3601 duplicate articles, and on the 4442 remaining studies, we performed a screening of titles and abstracts, reducing the selection to 503 articles coherent with our research criteria, which we read in their entirety. The final number of articles considered according to our inclusion criteria was 16 [23,31,40,65,66,67,68,69,70,71,72,73,74,75,76,77].

### 3.2. Study Characteristics and Design

Our selected studies were published in a long period ranging from 1985 to 2021. Concerning the geographical zone, the studies reported data only from Western countries: Europe (Finland, Germany, Great Britain, the Netherlands, Switzerland, and Sweden) and the United States. The average follow-up period of the studies is 7.21 (±6.83 SD) years, ranging from 1 to 30 years of collecting data. The designs of the studies are mostly of a prospective cohort; only two studies are case–control, and one is follow-up of a randomized control study.

Although the final number of articles included is 16 [23,31,40,65,66,67,68,69,70,71,72,73,74,75,76,77], only 15 populations were included in this review because two studies analyzed the same sample at different follow-up periods of 5 and 10 years, respectively [65,66], as shown in Table 1.

### 3.3. Study Samples

The sample sizes of the selected studies ranged between 25 and 16,448 persons [67,68]. The largest sample included consisted of the entire Swedish population in the period [68], from which emerged a number of 16,448 individuals affected by an eating disorder (Table 1). The study samples were mostly composed of females (from 89% to 100%); in some studies, the male population was deliberately excluded due to the low frequency of EDs in males.

Different types of eating disorders were reported in the study samples: Anorexia Nervosa (AN); Bulimia Nervosa (BN); Anorexia Nervosa-Restricting subtype (AN-R); Anorexia Nervosa Binge/Purge subtype (AN-BP); Eating Disorder Not Otherwise Specified (EDNOS); Other Specified Feeding and Eating Disorder (OSFED), including Binge Eating (BE) (Table 1).

### 3.4. Quantitative Analysis

In this systematic review, our meta-analysis addressed three quantitative outcomes: the first one, based on ED persistence, considered 11 studies; the second one, focused on the relapse percentage after a brief period of symptom improvement, based on the results of seven articles, and the third one concerning the mean duration of EDs analyzed seven studies. We performed a meta-analysis for three outcomes: eating disorder persistence at follow-up, relapse, and mean duration of the disorder.

### 3.5. ED Persistence

**Figure 2:**
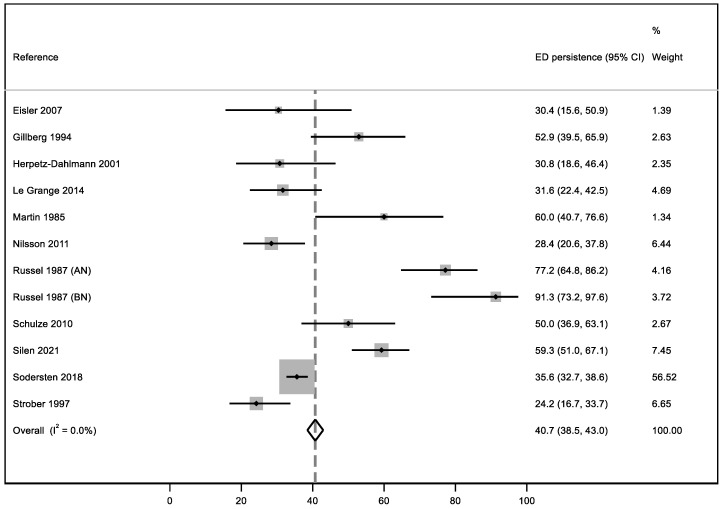
Meta-analysis of ED persistence in early adulthood [23,31,40,65,67,69,72,73,74,75,77].

In order to answer this research question, we considered 11 articles. To standardize and include all patient data, we included all individuals who did not achieve a complete remission at the end of the follow-up or at the end of the studies and/or people who still had slight symptomatology and/or subjects who were rated “Intermediate” or “Poor” according to the Morgan and Russell scale [34]. The percentage of patients still affected by an ED at the follow-up was 40.7% with null heterogeneity (I^2^ = 0%) (Figure 2). In Figure 2, we report the results of one study [31] divided into anorexia nervosa and bulimia nervosa (AN and BN) samples, as reported by the authors. The sample with the highest weighted average is the one analyzed by Södersten et al. [69].

### 3.6. ED Relapse

**Figure 3:**
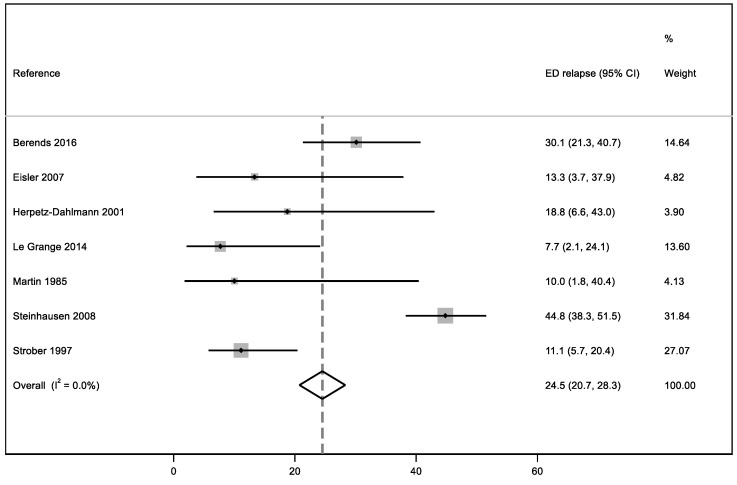
Meta-analysis of ED relapses in early adulthood [40,67,70,72,75,76,77].

We considered seven articles to calculate a meta-analysis, including patients showing both total and partial relapses; the final result of the study-weighted average of relapse is 24.5% with null heterogeneity (I^2^ = 0%) (Figure 3). The sample with the highest weighted average is the one analyzed by Steinhausen et al. [70].

### 3.7. ED Mean Duration

**Figure 4:**
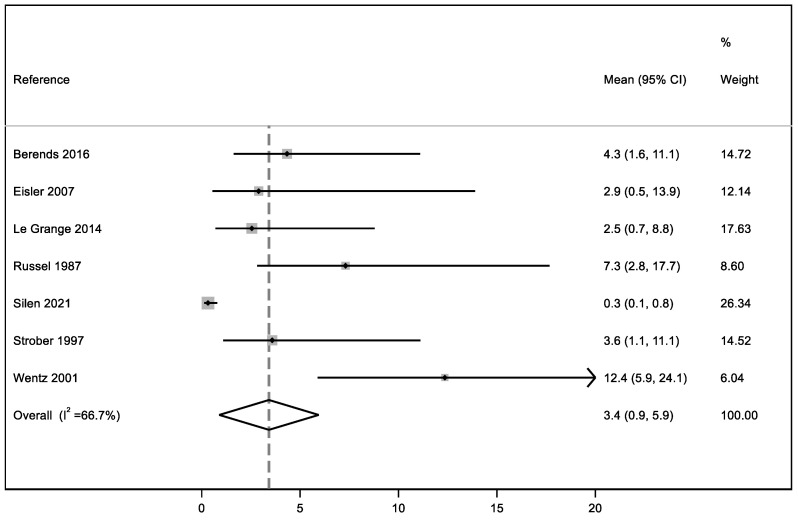
Meta-analysis of ED mean duration from its onset to early adulthood [31,40,66,72,74,75,76].

The mean duration of the disorder was analyzed in seven articles because in the remaining selected studies it was not possible to obtain information about this outcome due to a lack of data. In fact, for the patients who remained ill at the end of most of the studies, it was not possible to determine how long their illness had lasted because the time of ED onset had not been reported. Based on the seven articles included, the mean duration of the disorder is 3.4 years with moderate heterogeneity (I^2^ = 66.7%) (Figure 4).

### 3.8. Qualitative Outcomes

Our selected qualitative outcomes, psychiatric and medical comorbidities, substance use, and social–relational complications, were analyzed only in six studies, the only studies among our selection that reported these data [65,66,67,68,72,73]. We present a narrative synthesis of these outcomes because it was not possible to develop a meta-analysis on them due to the different instruments used to assess them and the lack of data homogeneity across the selected studies (Table 2).

### 3.9. Psychiatric Comorbidities

We reported psychiatric comorbidities in 5 of the 16 articles included:

(1) In Gillberg et al. [65], the authors noted an empathy deficit, diagnosed using the Dewey Social Awareness test, usually used to evaluate the presence of Asperger’s Syndrome [78]. Patients with an empathy deficit have difficulty in understanding others’ ideas, thoughts, and feelings and report a feeling of social ineptitude. This deficit was reported in 15 out of 51 individuals, already affected by anorexia nervosa [65].
(2) Le Grange et al. [72], using the DSM-IV (1990), noted mood disorders in 8 of 79 patients and, in the same sample, found that 12 individuals were affected by anxiety disorder.
(3) Martin et al. [67] in 1985 highlighted two comorbidities in 2 patients out of 22: one had a behavior disorder, while the other showed depression symptoms.
(4) Wentz et al. [66] study has the highest number of psychiatric comorbidities: the most common ones were dysthymia, obsessive-compulsive disorder, and anxiety disorder; to a lesser extent, the authors found that patients with EDs suffered from major depression, panic attacks, specific and social phobias, psychotic disorders, somatoform disorders, and tic disorders. One patient also developed substance abuse.
(5) Yao et al. [68], given the large sample size, found many comorbidities. The more common disorders were major depression, anxiety disorder, and substance abuse.

### 3.10. ED Medical Comorbidities and Death

The only medical comorbidity reported in the selected studies was amenorrhea, noted in four patients by Gillberg et al. [65]. The number of deaths is not very commonly reported; only two studies give us information about this data. Schulze et al. [73] reported two patients deceased, without describing the cause of death; Yao et al. [68] reported 48 deaths by suicide (27 individuals affected by anorexia nervosa and 11 with bulimia nervosa) in a very large sample of 16,448 individuals.

### 3.11. ED Social–Relational Complications

We only found one article that describes social complications correlated with eating disorders in patients who showed difficulties in the relationship with their family [65]: A total of 18 individuals affected by anorexia nervosa stated that the relationship with their relatives was unsatisfactory, and another 12 adolescents said that they had trouble affirming their independence from their household.

### 3.12. The Quality Studies Assessment

With the Newcastle-Ottawa Scale (NOS), the mean article score was 7.3 (Table 3). The lowest rate assigned was a 4, given to the study of Södersten et al. [69], and the highest score was a 9 given to two studies [40,68].

## 4. Discussion

Our results show that ED persistence is high (40.7%), referring to all individuals who, at the end of the study follow-up, still showed any kind of ED symptom, regardless of disorder severity, including therefore also cases in partial remission. This percentage is slightly lower compared with another study [79], which reported in a nine-year follow-up, a persistence percentage of 68.2% for anorexia nervosa and 31.8% for bulimia nervosa. Although from our selected studies, which analyzed mostly individuals affected by anorexia, we reported a lower mean persistence percentage, these data are rather alarming because they indicate a persistence of 10 years on average of EDs in a population of adolescents, who are in a crucial period of their development and their personal and social achievements. Relapse is an indicative outcome to fully understand the course of chronic illnesses, such as anorexia and bulimia nervosa. For this reason, we performed a meta-analysis to assess the percentage of the occurrence of this event. It is important to specify that the literature does not have unique criteria to define relapse [33], and for this reason, every article has a different standard to evaluate the recurrence of ED.

The relapse percentage analyzed in seven articles stands at 24.5%, in line with already published data in the literature. To support this result, we considered other authors’ research, reporting similar data: Bodell et al. [80] reported a percentage ranged between 20 and 30% for relapse; Carter et al. [81] observed a relapse percentage of 40% in their sample. Regarding the relapse, we can highlight that the highest risk for relapse occurs in the first year since the beginning of ED with slight variations, in accordance with many authors: Strober et al. [75] reported that the most critical period for relapse is the first 12 months, extended to 18 months according to Berends et al. [76]. Carter et al. [81] reported the most frequent relapses in a period ranged between the fourth and ninth month after the beginning of ED treatment. Relapse rarely occurs once total remission is achieved, as observed by Herpertz-Dahlmann et al. [77] who highlighted that 12 patients who achieved full remission did not relapse. The same results were reported by Eisler et al. [40], who highlighted a low percentage of relapses after the achievement of total remission (24 out of 26 never relapsed). Kordy et al. [36] highlighted that the risk of relapse is more elevated in patients in partial remission than in those whose remission has been fully achieved.

Our findings highlight that, although ED relapse may be present in nearly a quarter of people with these disorders, especially during the first year, the risk of recurrence became low in people who achieved full ED recovery, suggesting that EDs can be fully recovered. In this regard, some authors suggest the importance of hope, motivation, self-efficacy, and support from others in the recovery process also in enduring the ED, as both medical and recovery models suggest [82,83]. As concerns comorbidities, we found different psychiatric disorders correlated with anorexia and bulimia nervosa; among adolescents in the transition into adulthood, the most common disorders are anxiety disorders and major depression, in line with the current literature [45,46]. In this regard, it is necessary to underline that these observations were only reported in five articles among the studies selected, and only one reported social and medical complications.

The scarce number of articles reporting medical comorbidities could imply that, in the majority of cases, ED medical complications completely improve after the resolution of undernutrition. This aspect is supported by another systematic review focused on the medical complications of anorexia and bulimia nervosa, which underlines that early treatment rapidly reverting malnutrition reduces the risk for medical complications [84].

Another crucial observation reported by one of our studies regards social–relational complications related to EDs, indicating that the transition into adulthood of individuals affected by an ED is characterized by difficulties in the development of autonomy and independence from their parental family, with the risk of behavioral regression. We can hypothesize that the same family factors, which precede the onset of an ED, can maintain it, limiting the normal development of people with EDs. In fact, current family therapy is focused on improving emotional communication and developing skills of negotiation in order to foster a flexible attitude and autonomy among the family members [85].

A recent study has highlighted that the ED duration increases the risk of presenting early deficit in executive functions, in particular, in decision making, inhibitory control, and cognitive flexibility, which can undermine the autonomy and functioning capacity of people with EDs [86].

We underline the paucity of studies assessing the global functioning of people with EDs, which could represent an essential aspect to better evaluate the clinical course of these disorders. A study evaluated the impact of EDs on quality of life, whose score was found lower among individuals with EDs in comparison with the general population [87].

Another aspect, which is worth mentioning, concerns the different gender prevalence of EDs, which, in most cases, is in the female gender. Analyzing the study samples, we notice that the percentages of females are higher than males, in line with most published articles on this topic. A recent study conducted by Van Eeden et al. [16] reported an ED percentage of 4% in girls and only 0.1% in boys. Generally, authors tend to exclude male individuals from their studies, given their low rate of ED prevalence, leading, inevitably, to a limited investigation and poor knowledge of these disorders in the male population.

In the light of our results, we hope that the definition of occurrence, relapse, remission, and recovery of eating disorders can be clarified and unified in a shared definition in order to facilitate the scientific research and to obtain homogeneous results. To avoid a bias risk, we suggest including patients of all genders in the studies to obtain a more detailed view of these disorders. In fact, EDs among adolescent males are likely considerably underestimated although they are equally or perhaps even more quickly increasing than among females [88].

### Strengths and Limitations

The first strength of this review concerns the topic, because anorexia and bulimia nervosa follow-up from adolescence to early adulthood has not been exhaustedly reviewed. To collect the highest number of studies, we searched four databases, and for the same purpose, we did not impose any limit regarding geographical area or date of publication. Furthermore, regarding our outcomes, we included studies with both quantitative and qualitative results. In addition, the meta-analysis for both ED persistence and relapse showed overall consistent results between the included studies as indicated by the null heterogeneity, although the meta-analysis of mean duration showed moderate heterogeneity due to high variations of mean duration across studies. The intervention of a multiprofessional team is a strength. Each reviewer provided information based on their professional and experiential background; in different phases of the review, the team analyzed multiple aspects of the review, both minimizing bias and increasing reliability and truthfulness. Regarding the limitations, the first noteworthy one is the language: we reviewed only studies published in English or Italian. Another limitation is not investigating the grey literature, research published outside of commercial or academic journals, such as theses and dissertations, which would have allowed us to see other research in this field more extensively. Another limitation of this study is the analysis of all EDs as a whole, although bulimia, anorexia, and binge eating disorder are very complex disorders with specific clinical needs and different evolutions. In addition, the limited number of studies and lack of stratified data by ED type hamper the implementation of the analysis divided by ED diagnosis to explore the heterogeneity we found in the analysis of mean duration.

We defined the number of at least 20 patients as the inclusion criteria for the sample size, leaving behind the smaller studies conducted in the local setting and single clinical cases. The articles included in the review, like most of the studies found, reported data from Western countries, leaving out the populations of Africa, Latin America, and Asia. Other limitations concern the absence of a univocal definition of relapse, remission, and recovery, which led the authors of each study to develop their own evaluation method, and the lack of BMI values in several studies both at the beginning of the study and at the end of the follow-up, thus making it more difficult to unify the results Moreover, the lack of information regarding patient treatments could limit the generalizability of our results. Finally, some studies showed high drop-out rates, so we cannot entirely rule out the occurrence of selection bias for those studies.

## 5. Conclusions

In light of our results, we confirm that eating disorders have a chronic course with a persistence into early adulthood of 40.7%, a percentage of 24.5% relapse, and a mean duration of 3.4 years. Anxiety and depressive disorders are the most frequent comorbid disorders as well as reduced individual autonomy and independence from parental family. Among medical comorbidities, the only one observed was amenorrhea, strictly correlated with ED, particularly with anorexia.

## Figures and Tables

**Table 1:** Characteristics of population samples in the included studies.

Reference Year, Country	Study Design	Follow-Up (Years)	ED Mean Duration (Years)	Sample at Baseline (N)	Sample at Follow-Up (N)	Females N (%)	Age at Baseline (Years): Mean (SD)	BMI at Baseline and End of Follow-Up	ED Subtype N (%)
Berends et al. [76], 2016, The Netherlands	Cohort	1.5	3.6	83	83	83 (100)	17.9 (4.45)	Start: 16.3/16.4 ^a^ End: 19.9/20.4 ^a^	70 = AN-R (84.3) 13 = AN-BP (15.7)
Eisler et al. [40], 2007, UK	RCT follow-up	5	1.1	38	38	40 (100)	14 (1.6)	Start: 15.4 End: 19.8	40 = AN (100)
Gillberg et al. [65], 1994, Sweden	Case–control	5	NR	51	51	48 (94)	14.3 (13.9–14.7) ^b^	Start: 18.3 End:21.2	51 = AN (100)
Herpertz-Dahlmann et al. [77], 2001, Germany	Cohort	3–7	NR	44	39	35 (90)	14.9 (1.6)	Start: 14.3/15.7 ^c^ End: 20.2/24.6 ^c^	39 = AN (100)
Le Grange et al. [72], 2014, USA	Cohort	3.26	2	121	79	110 (91)	14.7 (1.6)	NR	72 = AN (91.1) 2 = BN (2.5) 5 = EDONOS (6.%)
Martin et al. [67], 1985, Canada	Cohort	5.1	NR	25	22	22 (88)	14.9 (2.12)	NR	18 = AN (72) 7 = BN (28)
Nilsson et al. [23], 2012, Sweden	Cohort	3	NR	165	102	102 (100)	15.86 (1.37)	Start: 18.1 End: NR	44 = AN (43) 12 = BN (12) 46 = EDNOS (45)
Russell et al. [71], 1985, UK	Cohort	9.5	NR	22	20	20 (100)	12.25	NR	17 = AN (85) 3 = BN (15)
Russell et al. [31], 1987, UK	Cohort	1	3.8	80	52	73 (91)	17.9 (6.4)	NR	57 = AN (71) 23 = BN (29)
Schulze et al. [73], 2010, Germany	Cohort	5.23	NR	52	52	52 (100)	15.5 (2.07)	Start: 14.74 End: 20.13	52 = AN (100)
Silèn et al. [74], 2021, Finland	Cohort	5	4.3	142	142	127 (89)	16.5 (2.9)	NR	46 = AN (31) 18 = BN (13) 6 = BE (4) 32 = OSFED (23) 42 = NS (30)
Södersten et al. [69], 2018, Sweden	Cohort	5	NR	12,854	5867	12,211 (95.4)	23.1 (8.9)	NR	3279 = AN (26) 3219 = BN (25) 3414 = BE (27) 2225 = OSFED (17) 717 = Others (7)
Steinhausen et al. [70], 2008, Switzerland	Cohort	8.3	NR	212	212	201 (95)	14.8 (1.7)	Start: 13.9 End: NR	212 = AN (100)
Strober et al. [75], 1997, USA	Cohort	12.5	2.4	95	95	85 (89.5)	Range: 12–17	Start: 14.1 End: NR	18 = AN-BP (18.9) 77 = AN-R (81.1)
Wentz et al. [66], 2001, Sweden	Case–control	10	10.2	88	51	48 (94)	AN: 13 (3) BN: 14 (6)	NR	37 = AN (73) 6 = BN (12) 8 = OSFED (16)
Yao et al. [68], 2016, Sweden	Cohort	30	NR	16,448	16,448	15,457 (94)	18.4 (4.0)	NR	8133 = AN (49) 3410 = BN (21) 4945 = Others (30)

^a^ Values reported for full/partial relapse group and no-relapse group, respectively; ^b^ 95% confidence interval reported; ^c^ Values for females and males, respectively. Abbreviations: AN, anorexia nervosa; BN, bulimia nervosa; AN-R, anorexia nervosa-restricting subtype; AN-BP, anorexia nervosa binge/purge subtype; BE, binge eating; EDONOS, eating disorder not otherwise specified; OSFED, other specified feeding and eating disorder; NR, not reported.

**Table 2:** Studies included in the qualitative analysis of medical and psychiatric comorbidities and substance use in EDs and associated social–relational complications.

Reference, Year	Psychiatric Comorbidities N (%)	Substance Use N (%)	Medical Comorbidities N (%)	Social–Relational Complications N (%)	Deaths N (%)
Gillberg et al. [65], 1994	17 (33%) Empathy Deficit	NR	4 (8%) Amenorrhea, Malnutrition	17 (34%) Social issues out of family 9 (18%) Family emancipation issues	NR
Le Grange et al. [72], 2014	8 (10%) Affective Disorders 2 (15%) Anxiety Disorders	NR	NR	NR	NR
Martin et al. [67], 1985	1 (4%) Behavior disorders 1 (4%) Depressive symptoms	NR	NR	NR	NR
Schulze et al. [73], 2010	NR	NR	26 (50%) Amenorrhea 6 (11.5%) Osteopenia	NR	2 (2%)
Wentz et al. [66], 2001	27 (53%) Major Depressive Episode 3 (6%) Bipolar Disorder 19 (37%): Dysthymia 2 (4%) Panic Attack 7 (14%) Specific Phobia 3 (6%) Social Phobia 18 (35%) Obsessive-compulsive Disorder 11 (21.5%) Generalized Anxiety Disorder 4 (8%) Psychotic Disorders 2 (4%) Somatoform Disorder	1 (2%) Substance use	7 (14%) Tic Disorders 6 (12%) Malnutrition 24 (50% of female sample) Amenorrhea	31 (61%) Social issues 18 (35%) Work issues	NR
Yao et al. [68], 2016	5247 (31.9%) Major Depressive Episode 3742 (22.8%) Anxiety Disorders	1731 (10.5%) Substance use	NR	NR	48 (0.29%)

Abbreviation: NR, not reported.

**Table 3:** Risk of bias assessment of included studies using the Newcastle–Ottawa Scale.

Cohort Studies	Selection	Comparability	Outcome	Total
Berends et al. [76], 2016	3	1	3	7
Eisler t al [40], 2007	4	2	3	9
Herpertz-Dahlmann et al. [77], 2001	3	2	3	8
Le Grange et al. [72], 2014	3	1	3	7
Martin et al. [67], 1985	3	1	3	7
Nilsson et al. [23], 2011	2	2	3	7
Russell et al. [71], 1985	3	1	3	7
Russell et al. [31], 1987	3	2	3	8
Schulze et al. [73], 2010	3	2	3	8
Silèn et al. [74], 2021	3	2	3	8
Södersten et al. [69], 2018	2	1	1	4
Steinhausen et al. [70], 2008	3	2	2	7
Strober et al. [75], 1997	3	2	2	7
Yao et al. [68], 2016	4	2	3	9
**Case–Control Studies**	**Selection**	**Comparability**	**Exposure**	**Total**
Gillberg et al. [65], 1994	3	1	3	7
Wentz et al. [66], 2001	3	1	3	7

## Data Availability

The data presented in this study are available in the article.
